# Local Expression of Epigenetic Candidate Biomarkers of Adolescent Idiopathic Scoliosis Progression

**DOI:** 10.3390/ijms26178453

**Published:** 2025-08-30

**Authors:** Simona Neri, Alberto Ruffilli, Elisa Assirelli, Marco Manzetti, Giovanni Viroli, Matteo Traversari, Marco Ialuna, Susanna Naldi, Jacopo Ciaffi, Francesco Ursini, Cesare Faldini

**Affiliations:** 1 Medicine and Rheumatology Unit, IRCCS Istituto Ortopedico Rizzoli, 40136 Bologna, Italy; susanna.naldi@ior.it (S.N.); jacopo.ciaffi@ior.it (J.C.); francesco.ursini@ior.it (F.U.); 2 1st Orthopaedic and Traumatologic Clinic, IRCCS Istituto Ortopedico Rizzoli, 40136 Bologna, Italy; alberto.ruffilli@ior.it (A.R.); marco.manzetti@ior.it (M.M.); matteo.traversari@ior.it (M.T.); marco.ialuna@ior.it (M.I.); cesare.faldini@ior.it (C.F.); 3 Department of Biomedical and Neuromotor Sciences (DIBINEM), Alma Mater Studiorum University of Bologna, 40126 Bologna, Italy

**Keywords:** Adolescent Idiopathic Scoliosis (AIS), AIS progression, epigenetics, biomarkers, local gene expression

## Abstract

Adolescent idiopathic scoliosis (AIS) is a multifactorial disease with environmental and genetic components. AIS clinical management is complicated by the lack of reliable predictive markers of progression. Recent studies have highlighted a potential role for epigenetic mechanisms in disease progression. However, most findings derive from peripheral blood analyses, with little data available on musculoskeletal tissues directly affected by AIS. Given the tissue-specific nature of epigenetic regulation, validating blood-based biomarkers in disease-relevant tissues is essential. We performed a comparative multi-gene RT-qPCR analysis, arranged in a custom array format, to assess the local expression of candidate epigenetically regulated genes associated with AIS progression across bone, paravertebral muscle, spinal ligament, and peripheral blood, all collected from the same patients. Tissue- and gene-specific expression patterns were observed, supporting the presence of local regulatory mechanisms. Peripheral blood expression of *HAS2*, *PCDH10*, *H19*, *ADIPOQ*, *ESR1*, *GREM1*, *SOX9*, *FRZB*, *LRP6*, and *FBN1* resembled bone expression, while *PITX1*, *CRTC1*, *APC*, *CTNNB1*, *FZD1*, and *AXIN1* reflected muscle and ligament; *WNT1* reflected only muscle. In contrast, *GREM1* and *SOX9* were expressed only in muscle and ligament and *FGF4* and *NPY* only in muscle, suggesting limited systemic biomarker potential. Compared to non-AIS tissues, AIS samples showed downregulation of *PCDH10* and *FBN2* in bone and *CRTC1*, *FRZB*, *LRP6*, and *MSTN* in muscle. *WNT1* and *WNT10* were upregulated in muscle and *FBN1* in ligament. In conclusion, the results highlight differential gene expression across AIS tissues, supporting tissue-specific regulation in some of the genes analyzed. Only a subset of markers exhibited blood expression patterns that reflected those in specific tissues, suggesting that certain blood biomarkers may act as surrogates for distinct tissue compartments. These results lay the groundwork for future DNA-based studies to confirm the epigenetic nature of this regulation and to identify reliable biomarkers for AIS progression.

## 1. Introduction

Adolescent idiopathic scoliosis is a complex three-dimensional deformity of the spine, with different grades of involvement of the frontal, sagittal, and axial planes. It affects 2–3% of the adolescent population, mainly females [1]. After receiving an AIS diagnosis, which is based on clinical examination and radiographic evaluation, patients require different management (from observation to orthotic treatment and surgical correction) based on the curve magnitude at diagnosis and progression potential. While the Cobb angle is a straightforward tool in initial curve magnitude measurement, it might be more challenging to assess each patient’s risk of curve progression at diagnosis. The identification of reliable curve progression indicators is essential to prevent incorrect clinical management that could deprive patients of adequate treatments or expose others to unnecessary ones. Many clinical and radiographic parameters are widely accepted as predictors of scoliosis progression, but they have all shown limited prediction power. Considering that clinical features appear inadequate to predict disease evolution, the study and identification of molecular factors associated with scoliosis progression could be of crucial relevance in clinical practice. Molecular biomarkers may offer valuable insights into the pathogenic mechanisms underlying AIS, which remain largely unknown. Beyond advancing our understanding of disease biology, molecular profiling could support the stratification of patients into more homogeneous clinical and biological subgroups, each with a distinct risk of progression [2]. This, in turn, may contribute to the development of more accurate prognostic models and pave the way for targeted and personalized therapeutic strategies [3]. Epidemiological and genetic studies have indicated AIS as a polygenic disease and investigated genetic and epigenetic factors linked to an increased risk of scoliotic curve onset. Several loci associated with AIS susceptibility were identified and evaluated in different ethnic groups, although the value of AIS susceptibility in clinical practice is limited [4,5].

Less is known about the role that genetics and epigenetics play in disease progression and prediction, which is crucial for disease management. To date, single-nucleotide polymorphisms (SNPs) in 15 genes have been linked to progressive AIS: ER, IGF1, MATN1, CALM1, TPH-1, NFT3, IL-17RC, LBX1, LAPTM4B, BNC2, FBN1/, TIMP 2, SOX9, CDH7, and MIR4300 [6,7,8]. Combined panels of these SNPs were built to obtain predictive algorithms to discriminate patients with low or high progression risk [4]. Nevertheless, the identified genetic tests displayed insufficient evidence to guide therapeutic choices or showed disappointing validation in different ethnicities [9,10,11,12,13].

More recently, there is a growing body of research supporting a role for epigenetics (including DNA methylation, chromatin structure, and non-coding RNAs) in disease progression [14,15,16,17,18,19], including wide methylation studies in monozygotic twins [15]. *COMP* (cartilage oligomeric matrix protein) [18], *PITX1* (pituitary homeobox 1) [17], and *PCDH10* (protocadherin-10) [19] gene methylation were correlated with AIS curve severity. Conversely, *HAS2* (hyaluronan synthase 2) [15], *WNT* (wingless-type MMTV integration site family) genes, and *NPY* (neuropeptide Y) showed demethylation associated with AIS progression [16], and wide methylation studies in monozygotic twins have highlighted an association between diffuse hypomethylation and disease severity [15,16]. Moreover, differential expression of selected miRNAs has been associated with disease grade [14,20,21].

Several studies have proposed circulating biomarkers, predominantly including miRNAs, as potential tools for AIS progression prediction, with a focus on their accessibility [3,22]. Multi-omics approaches have begun to identify potential biomarker signatures that could improve prognostic accuracy beyond traditional clinical parameters [23].

Most of the studies have been performed on DNA from peripheral blood, while only a few reports have analyzed musculoskeletal tissues involved in the disease, primarily paravertebral and paraspinal muscles [24], and to the best of our knowledge, no studies have analyzed and compared the different tissues involved locally. Since epigenetic regulation of gene expression is tissue-dependent [25,26,27], it cannot be ruled out that candidate epigenetic biomarkers behave differently in musculoskeletal tissues compared to peripheral blood cells. To identify the mechanisms underlying disease progression and find reliable predictive biomarkers, it is therefore mandatory to validate data obtained from peripheral blood directly against the tissues affected by the disease.

From this perspective, we performed an exploratory analysis by characterizing the local gene expression of candidate epigenetic factors that have been shown in recent literature as possibly involved in AIS progression. To effectively link the degree of activity of these genes to the tissues directly involved in AIS onset and progression and to assess whether peripheral blood can represent a surrogate of diseased tissue behavior, we employed an original protocol developed for this purpose [28]. This protocol involved a comprehensive analysis of AIS-related tissues—including bone facets, paravertebral muscles, paraspinal ligament, and peripheral blood—all collected from the same patients. The goal was to conduct an in-depth local gene expression analysis, serving as a prerequisite for identifying potential epigenetic regulatory mechanisms within affected tissues. To further refine the analysis, the concave and convex sides of the spinal curvature were examined separately—in the bone and muscular compartments—to distinguish gene activity based on anatomical localization. We observed a local regulation of gene expression that appeared to be tissue-dependent and gene-specific. These findings support the rationale for subsequent analyses of DNA to demonstrate the actual presence of epigenetic control mechanisms, such as differential methylation.

## 2. Results

### 2.1. Gene Expression Profiling Across Musculoskeletal Tissues

**Figure 1:**
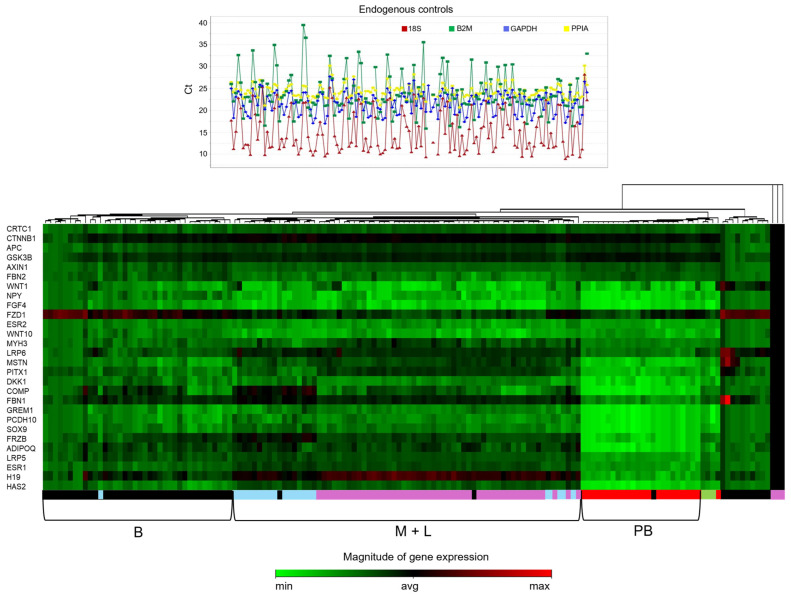
Local gene expression analysis of 28 putative epigenetic markers of AIS progression. **Upper panel**: Variability in the expression levels (Ct = cycle threshold) of *18S* (red), *B2M* (green), *GAPDH* (blue), and *PPIA* (yellow) housekeeping genes measured across all tissue samples included in the study: bone, muscle, ligament, and peripheral blood. *GAPDH* and *PPIA* are less variable than the other two. **Lower panel**: Heatmap and unsupervised hierarchical clustering of the expression levels of 28 genes (*Y*-axis) across bone (B), muscle (M), ligament (L), and peripheral blood (PB) tissue samples (*X*-axis). Colors reflect deviation from the mean ΔCt value, with red indicating higher and green indicating lower expression levels. Below the heatmap, the tissue types of the samples are indicated by colors (black for bone, fuchsia for muscle, light blue for ligament, and red for peripheral blood), showing that samples belonging to the same tissue type are predominantly similar and clustered together. Further below, the three primary tissue clusters are also indicated.

Twenty-eight protein-coding genes were analyzed for their local expression in AIS tissues. Expression data were normalized to the reference GAPDH and PPIA genes, selected as the most stable and consistent. As shown in Figure 1 (upper panel) and Supplementary Table S1, their expression levels exhibited a notably narrower range of variability compared to 18S and B2M, supporting their selection as the most stable reference genes for normalization.

In the lower panel of Figure 1, cumulative gene expression is presented as a heatmap. Using unsupervised hierarchical cluster analysis, it was observed that expression profiles were generally consistent among samples of the same tissue type, which clustered closely together, whereas distinct tissue types exhibited divergent expression patterns. Notably, samples from the same tissue type exhibited greater similarity to each other than to samples from the same patient but different tissues, highlighting the predominance of tissue-specific gene expression regulation. Clustering primarily reflected the tissue of origin, with the dendrogram revealing three major clusters: (1) a muscle–ligament cluster subdivided into one muscle subgroup and two distinct ligament subgroups, indicating close transcriptional relationships; (2) a bone cluster; and (3) a peripheral blood cluster, forming a distinct homogeneous group (Figure 1). Notably, a ligament subgroup and a bone subgroup showed closer association with the blood cluster than with the primary muscle–ligament cluster, suggesting transitional expression profiles and possible sample-specific variability. Additionally, three muscle samples were positioned distantly from all clusters, possibly reflecting biological variability; however, their distinct positioning requires further investigation.

**Figure 2:**
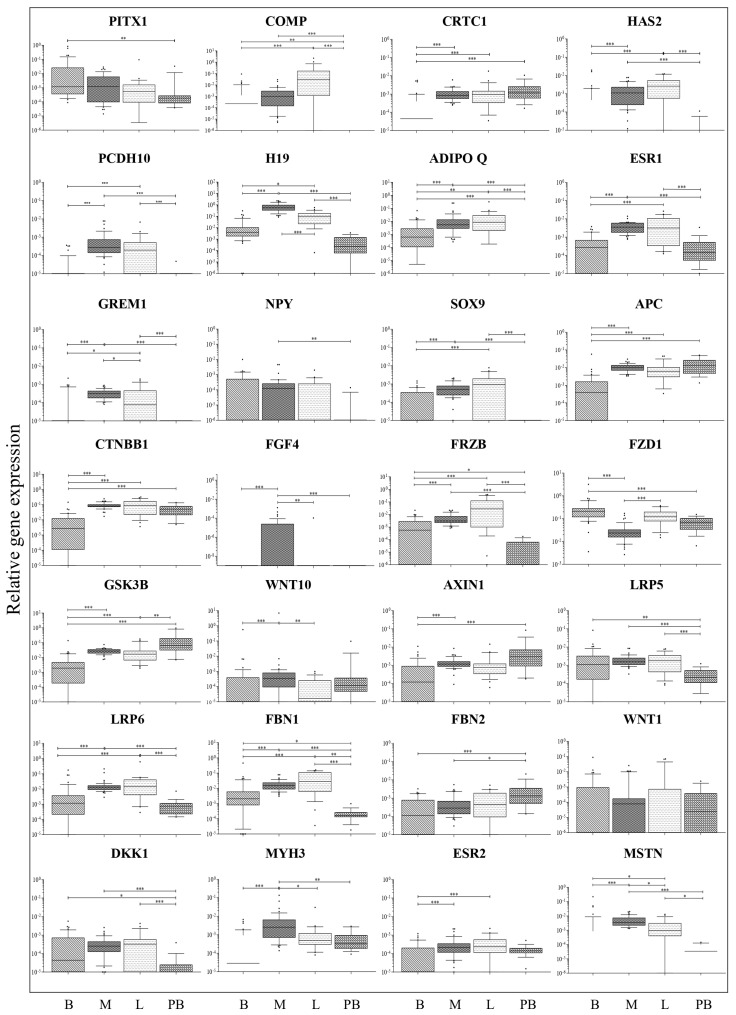
Local gene expression analysis in AIS tissues. Relative gene expression of 28 putative epigenetic markers of AIS progression in bone facets (B), intervertebral muscle (M), spinal ligament (L), and peripheral blood (PB) of 21 donors with AIS. Gene expression levels (relative to *PPIA* and *GAPDH* housekeeping genes) are shown as medians. Boxes indicate 10% to 90% percentiles, whiskers represent min to max values, and dots represent outliers. Comparisons among different tissue types were made using the Kruskal–Wallis test followed by Dunn’s correction for multiple comparisons. * = *p* ≤ 0.05; ** = *p* ≤ 0.01 *** = *p* ≤ 0.001.

Individual gene expression in the different AIS tissues (bone, muscle, ligament, and blood, compared with each other) is shown in Figure 2. Many genes showed very low expression or were exclusively expressed in some tissues and absent in others. *FGF4*, *NPY*, and *WNT1* were not expressed in nearly all samples of all tissues (except for muscle), both in AIS and non-AIS cases. *GREM1* and *SOX9* were not expressed in any blood samples; they were detected in 16% and 34% of bone samples, respectively, with median relative expression levels of 0.0 for both genes. Conversely, these genes were expressed in all muscle samples (median relative expression levels: 0.00047 for *GREM1* and 0.000521 for *SOX9*) and in the majority of ligament samples (71 and 76%, respectively) with median expression levels of 0.00008 for *GREM1* and 0.00030 for *SOX9.*

Notably, muscle and ligament shared similar expression profiles, with no significant differences observed in the expression levels of several genes (*CRTC1*, *H19*, *ADIPOQ*, *ESR1*, *GREM1*, *SOX9*, *APC*, *CTNBB1*, *FRZB*, *GSK3B*, *LRP6*, *ESR2*, and *MSTN*) (Figure 2), consistent with hierarchical clustering that predominantly groups these two tissues together. Conversely, the genes *HAS2*, *PCDH10*, *H19*, *ADIPOQ*, *ESR1*, *GREM1*, *SOX9*, *FRZB*, *LRP6*, and *FBN1* were significantly less expressed in bone than in muscle and ligament (Figure 2). Notably, *FZD1* was the only gene found to be more highly expressed in bone than in muscle tissue, diverging from the general trend observed for the other genes.

The same analysis of 28 genes was performed on tissue samples from non-AIS individuals (degenerative spinal disease) to provide a reference for tissue-specific gene expression under a different condition (Supplementary Figure S1). For some genes, expression patterns exhibited a similar overall trend in AIS and non-AIS tissues (*CRTC1*, *HAS2*, *H19*, *ADIPOQ*, *ESR1*, *GREM1*, *SOX9*, *APC*, *CTNNB1*, *FGF4*, *FRZB*, *FZD1*, *AXIN1*, *LRP5*, *LRP6*, *FBN2*, *DKK1*, and *MSTN*). However, for other genes, differences were observed in the relative expression levels across the various tissues, indicating altered tissue-specific expression relationships between the two conditions (*COMP*, *PCDH10*, *NPY*, *GSK3B*, *WNT10*, *FBN1*, *WNT1*, *MHY3*, and *ESR2*).

Compared to non-AIS tissues of the same type, AIS tissues typically showed lower gene expression levels: *PCDH10* and *FBN2* in AIS bone (*p* = 0.0176 and *p* = 0.0128, respectively); *CRTC1*, *FRZB*, *LRP6*, and *MSTN* in AIS muscle (*p* = 0.0410, *p* = 0.0324, *p* = 0.0069, and *p* = 0.0276, respectively); *PCDH10* in AIS ligament (*p* = 0.0259); and *APC*, *CTNBB1*, *FRZB*, *FBN2*, and *ESR2* in peripheral blood (*p* = 0.0034, *p* = 0.0009, *p* = 0.0343, *p* = 0.0224, *p* = 0.0034, and *p* = 0.0019, respectively). This suggests a possible inhibition of gene expression in AIS tissues. In only a few cases, AIS tissues showed higher expression than non-AIS tissues: *WNT1* and *WNT10* in AIS muscle (*p* = 0.0424 and *p* = 0.0049, respectively); *FBN1* in AIS ligament (*p* = 0.0010); and *FZD1* in AIS peripheral blood (*p* = 0.0019).

### 2.2. Comparison Between Musculoskeletal Tissues and Peripheral Blood

For a molecule to serve as a biomarker, it should be easily accessible—such as through a blood sample—and reflect the molecular behavior of the tissues of interest. We therefore investigated whether gene expression patterns in blood mirror those observed in the target tissues.

AIS musculoskeletal tissues almost always differed significantly from peripheral blood, suggesting caution in interpreting blood results as a surrogate for what happens in other tissues. Several genes showed different expression in peripheral blood compared to bone (*PITX1*, *COMP*, *CRTC1*, *HAS2*, *ADIPO Q*, *APC*, *CTNBB1*, *FRZB*, *FZD1*, *GSK3B*, *AXIN 1*, *LRP5*, *FBN1*, *FBN2*, and *DKK1*); muscle (*COMP*, *CRTC1*, *HAS2*, *PCDH10*, *H19*, *ADIPO Q*, *ESR1*, *GREM1*, *NPY*, *SOX9*, *FRZB*, *LRP5*, *LRP6*, *FBN1*, *FBN2*, *DKK1*, *MYH3*, and *MSTN1*); and ligament (*COMP*, *CRTC1*, *HAS2*, *PSCDH10*, *H19*, *ADIPO Q*, *ESR1*, *GREM1*, *SOX9*, *FRZB*, *GSK3B*, *LRP5*, *LRP6*, *FBN1*, *DKK1*, and *MSTN*) (Figure 2).

The subset of genes (*HAS2*, *PCDH10*, *H19*, *ADIPOQ*, *ESR1*, *GREM1*, *SOX9*, *FRZB*, *LRP6*, and *FBN1*) that were expressed at lower levels in bone compared to muscle and ligament also showed reduced expression in peripheral blood relative to these tissues. This observation suggests that, for these genes, blood expression patterns may reflect bone-specific regulatory mechanisms.

In other instances, peripheral blood gene expression more closely resembled that of muscle and ligament rather than bone (e.g., *PITX1*, *CRTC1*, *APC*, *CTNNB1*, *FZD1*, and *AXIN1*).

Finally, in some instances, the peripheral blood behaved differently from all other tissues (COMP, ADIPOQ, FRZB, LRP5, FBN1, and DKK1). These results indicate that specific candidate biomarkers are likely to act as surrogates for specific, rather than all, tissue compartments.

### 2.3. Comparison Between Convex and Concave Tissue Compartments

**Figure 3:**
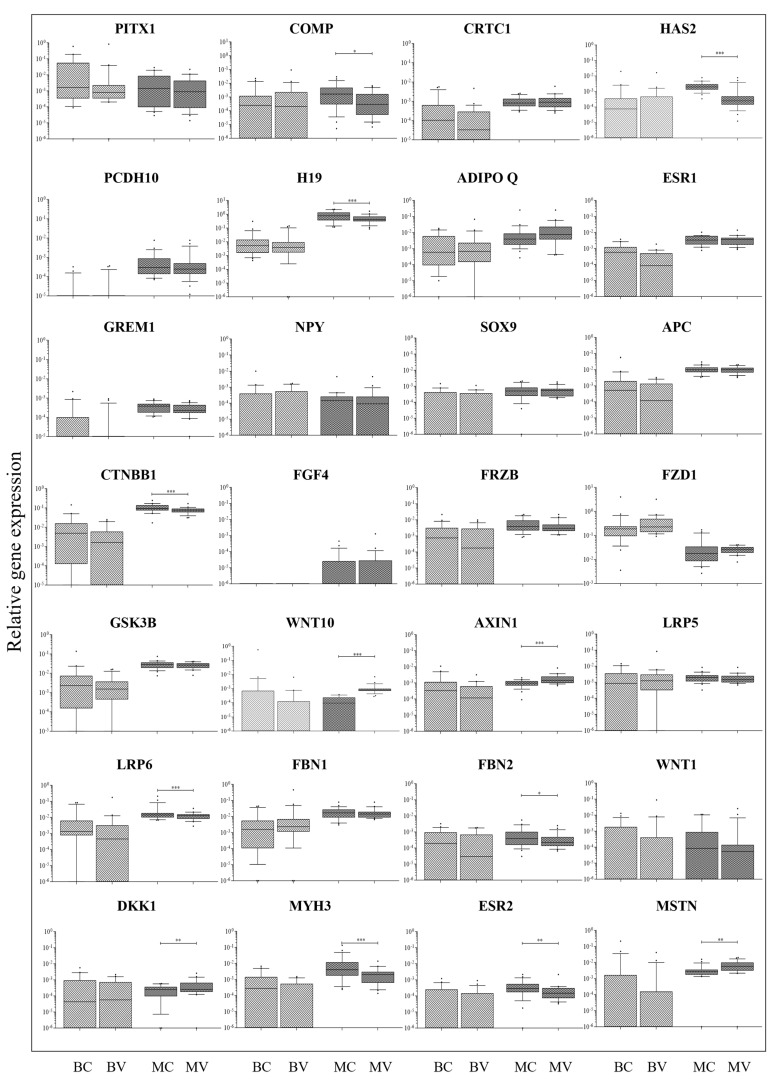
Local gene expression analysis in AIS tissues. Relative gene expression of 28 putative epigenetic markers of AIS progression in convex and concave bone facets (BV and BC, respectively) and in convex and concave paravertebral muscle (MV and MC, respectively) of 21 donors with AIS. Gene expression levels (relative to *PPIA* and *GAPDH* housekeeping genes) are shown as medians. Boxes indicate 10% to 90% percentiles, whiskers indicate min to max values, dots indicate outliers. Comparisons were made by the Wilcoxon matched-pairs test. * = *p* ≤ 0.05; ** = *p* ≤ 0.01 *** = *p* ≤ 0.001.

To explore possible differential regulation in relation to curve position within the same tissue type, the convex and concave sides of AIS bone and muscle tissues were further compared (Figure 3). All the genes were similarly expressed in bone regardless of the convex or concave compartment. Conversely, 12 genes showed differential expression between concave and convex muscle, with 8 of them upregulated in convex muscle (*COMP*, *p* = 0.0391; *HAS2*, *p* < 0.0001; *H19*, *p* = 0.0001; *CTNBB1*, *p* = 0.0009; *LRP6*, *p* = 0.0029; *FBN2*, *p* = 0.0115; *MYH3 p* = 0.0007, and *ESR2*, *p* = 0.0059) and 4 in concave muscle (*WNT10*, *p* = 0.0002; *AXIN1*, *p* = 0.0007; *DKK1*, *p* = 0.0029; and *MSTN*, *p* = 0.0011).

### 2.4. miRNA Expression Analysis

**Figure 4:**
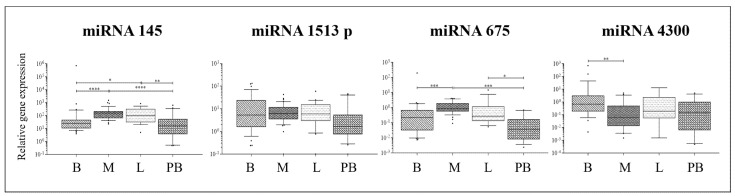
Local gene expression analysis in AIS tissues. Relative gene expression of 4 miRNAs possibly associated to AIS progression in bone facets (B), intervertebral muscle (M), spinal ligament (L), and peripheral blood (PB) of 21 donors with AIS. Gene expression levels (U6 miRNA used as reference) are shown as medians. Boxes indicate 10% to 90% percentiles, whiskers indicate min to max values, and dots indicate outliers. Comparisons among different tissue types were made by Kruskal–Wallis test followed by Dunn’s correction for multiple comparisons. * = *p* ≤ 0.05; ** = *p* ≤ 0.01 *** = *p* ≤ 0.001; **** = *p* ≤ 0.001.

Only two out of the four analyzed miRNAs showed different expression in AIS tissues compared to peripheral blood; miRNA-145 was more expressed in bone, muscle, and ligament compared to peripheral blood, while miRNA-675 was more expressed in muscle and ligament compared to peripheral blood (Figure 4). Therefore, the expression patterns observed in musculoskeletal tissues were reflected in the blood for miRNA-151-3p and miRNA-4300. In contrast, miRNA-145 and miRNA-675 showed expression profiles in blood that resembled those in bone but differed from those in muscle and ligament. These findings again support the hypothesis that candidate biomarkers may serve as surrogates for specific tissue compartments.

Compared to non-AIS tissues of the same type (whose gene expression data are shown in Supplementary Figure S2), AIS tissues typically showed lower miRNA expression levels, frequently reaching statistical significance: miRNA-145 and miRNA-675-5p were less expressed in AIS vs. non-AIS bone (*p* = 0.045 and *p* = 0.0207, respectively); miRNA-4300 was less expressed in AIS vs. non-AIS muscle (*p* = 0.0189); and miRNA-145, 151-3p, 675-5p, and 4300 were less expressed in AIS vs. non-AIS peripheral blood (*p* = 0.045 and *p* = 0.0207, respectively).

**Figure 5:**
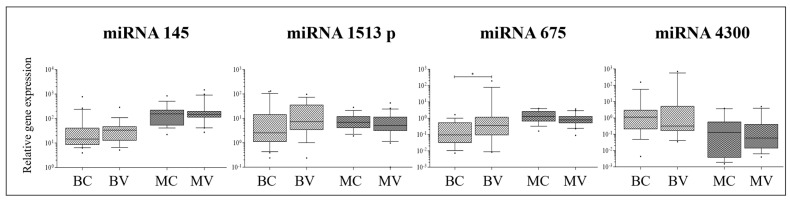
Local gene expression analysis in AIS tissues. Relative gene expression of 4 miRNAs possibly associated to AIS progression in convex and concave bone facets (BV and BC, respectively) and in convex and concave paravertebral muscle (MV and MC, respectively) of 21 donors with AIS. Relative gene expression levels (U6 miRNA used as reference) are shown as medians. Boxes indicate 10% to 90% percentiles, whiskers indicate min to max values, and dots indicate outliers. Comparisons were made by the Wilcoxon matched-pairs test. * = *p* ≤ 0.05.

Except for miRNA-675-5p, which was less expressed in convex bone, miRNAs did not exhibit a distinct distribution in the convex and concave compartments of muscle and bone (Figure 5).

### 2.5. Correlation Between Gene Expression and Clinical Parameters

**Figure 6:**
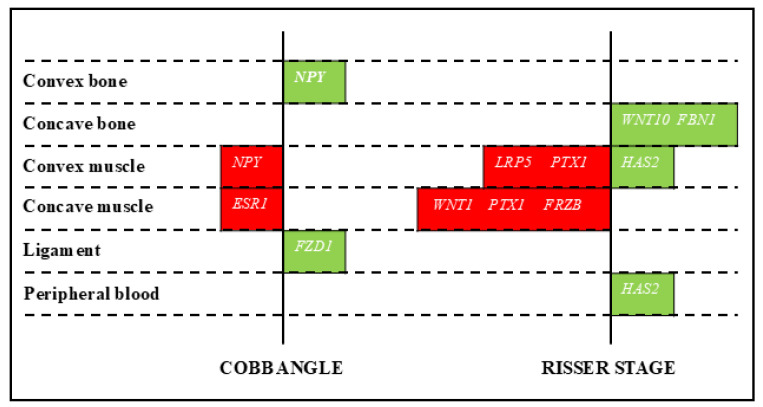
Correlations between local gene expression and Cobb angle (**left graph**) or Risser stage (**right graph**). Colored boxes indicate the specific genes showing positive (green) or negative (red) correlations with the clinical parameter.

Correlations between expression levels of the putative epigenetic biomarkers and clinical parameters of disease severity were also investigated. Some positive and negative correlations with Cobb angle and Risser stage were identified (Figure 6), although without consistency between the two scores. No correlations between scores and miRNAs were detected.

## 3. Discussion

The involvement of epigenetic mechanisms in AIS pathogenesis and progression has been widely documented [6,29], generating hope of identifying reliable biomarkers to guide therapeutic choices and allow for the development of innovative personalized prevention and treatment strategies.

While promising, available studies are mostly based on peripheral blood samples, and their relevance to local tissue dynamics remains largely unexplored. This preliminary and exploratory analysis performed across various AIS tissues—bone, muscle, ligament, and blood—responds to this gap by evaluating the expression of genes previously proposed as epigenetically regulated in AIS directly within affected musculoskeletal tissues.

This allowed for the characterization of the overall expression landscape within musculoskeletal compartments of individuals with AIS. Data were compared to peripheral blood from the same patients, offering a meaningful internal control. The approach used allows for a unique paired analysis that strengthens our interpretation of disease- and tissue-specific expression changes and reflects the potential to identify accessible circulating biomarkers that mirror local tissue activity in patients with AIS.

While we did not directly assess epigenetic modifications, our aim was to establish whether expression patterns in affected tissues support further investigation at the epigenetic level. The use of GAPDH and PPIA as housekeeping genes for relative quantification in gene expression analysis was validated by their consistent expression across different tissue types. This consistency is crucial for ensuring the reliability of comparative gene expression studies [30,31].

The tissue-specific nature of gene expression was evident in our study, with samples of the same tissue type showing higher similarity to each other than samples from different tissue types of the same patient, corroborating previous studies emphasizing the importance of tissue-specific regulatory mechanisms in gene expression [32]. Distinct patterns were observed across tissues, with certain genes showing preferential or exclusive expression in specific compartments (such as in bone compared to muscle and ligament). This underscores the importance of accounting for tissue context in AIS-related gene expression analyses and suggests the involvement of tissue-specific epigenetic regulatory mechanisms, which warrant further investigation and confirmation at the DNA level. These observations align with the known differential expression patterns of these genes in various physiological contexts [33].

In accordance with the identification of a characteristic gene expression pattern in AIS patients [34], we detected differential expression trends in AIS compared to non-AIS tissues. In general, AIS tissues showed a trend toward lower expression levels of several genes, pointing to a potential epigenetically mediated inhibition of gene expression in AIS, which may contribute to the pathophysiology of the condition. Notably, genes such as *PCDH10* and *FBN2* were downregulated in AIS bone, and *CRTC1*, *FRZB*, *LRP6*, and *MSTN* were downregulated in AIS muscle. Similar findings have been reported by Zhuang [35] and Hui [36], who identified downregulation of specific genes in AIS tissues compared to controls. Conversely, only a few genes were upregulated in AIS tissues, including *WNT1* and *WNT10* in AIS muscle and *FBN1* in AIS ligament. This differential expression could be indicative of compensatory mechanisms or alternative regulatory pathways activated in response to the pathology, in accordance with the observation of similar patterns of gene regulation in AIS tissues [37].

Differences in gene expression between convex and concave tissue compartments were observed only in muscle and not in bone and were mostly represented by reduced expression in the concave compartment. This suggests a relatively uniform molecular profile within bone tissue, regardless of curve position. In contrast, the observed differential expression of multiple genes in muscle indicates localized molecular adaptations that may be related to biomechanical or pathological asymmetries in AIS. It remains to be established whether this is a cause of the pathology or more likely a consequence.

Our findings may contribute to the identification of pathway-level dysregulations in AIS, thereby informing future studies on therapeutic modulation of specific signaling cascades, such as the Wnt pathway, known to play a central role in skeletal development and muscle differentiation. Specifically, WNT10, a canonical Wnt ligand involved in osteogenesis and myogenesis, was overexpressed in the convex paraspinal muscle of patients with AIS compared to concave muscle and to ligament and bone. Its increased expression may reflect compensatory mechanisms aiming to maintain tissue homeostasis in response to asymmetric mechanical loading or altered cellular signaling within the paravertebral muscles. This is consistent with previous reports by Zhu et al. [12] and Xu et al. [38], which reported significant asymmetric expression of WNT/β-catenin pathway components in bilateral paraspinal muscles and by studies showing that WNT signaling contributes to muscle regeneration and remodeling under stress conditions [39], thus unveiling the potential role of Wnt/beta-catenin pathway in the development of AIS.

This study also investigated miRNA expression, noting significant differences in miRNA-145 and miRNA-675-5p between AIS and non-AIS bone tissues and miRNA-4300 in muscle. The general trend in musculoskeletal tissues was mirrored in the blood, except for miRNA145. These findings highlight the complexity of miRNA regulation in AIS and suggest that while some miRNAs may serve as potential biomarkers, their tissue-specific expression patterns must be carefully considered. Similar trends have been reported in studies examining the roles of miRNAs in AIS [22,36].

A critical aspect of most AIS epigenetics studies is the use of peripheral blood as a surrogate for tissue-specific gene expression [15,16,17,18]. While blood is an easily accessible source, it does not necessarily reflect what occurs in other tissues. Indeed, epigenetic regulation affects gene expression and is tissue-dependent [25,26,27]. Accordingly, our findings revealed substantial discrepancies in gene expression between blood and musculoskeletal tissues, underscoring the limitations of using blood-based markers as a universal surrogate. Rather, data suggest that blood may reflect gene expression in specific tissues or for specific genes only, with no possibility of generalization and only after the validation of a direct correspondence within specific biological contexts. This aligns with the findings of Cheng [40], who reported similar limitations in using blood markers for AIS diagnosis.

Finally, this study explored correlations between gene expression levels and clinical parameters of AIS severity. While some correlations were identified, there was a lack of consistency across different scores, possibly due to the limited sample size and indicating the need for larger cohorts, enabling stratification by curve severity. This inconsistency is in line with Newman [41], who highlighted the challenges in identifying reliable biomarkers for AIS severity due to the complex and multifactorial nature of the disease.

Although some epigenetic markers identified here have been implicated in other neuromusculoskeletal conditions, our findings are specific to AIS. Future studies may investigate potential overlaps, but only the methodological framework applied here can be broadly extended beyond AIS.

While this study represents, to our knowledge, the first comparative analysis of gene and miRNA expression across multiple AIS-related tissues from the same individuals, it does have some limitations that warrant consideration. The use of RT-qPCR, though appropriate for this initial investigation, can pose challenges in normalization, particularly when comparing different tissue types. Future studies leveraging transcriptome-wide RNA sequencing will undoubtedly enhance data robustness and open new opportunities for broader biomarker discovery. The limited sample size and the absence of age-matched non-AIS controls somewhat constrain the generalizability of our findings. Furthermore, direct validation of epigenetic mechanisms at the DNA level remains an important next step. Despite these limitations, this preliminary work provides a valuable foundation and is intended to stimulate larger-scale studies aimed at validating and extending these findings.

## 4. Materials and Methods

### 4.1. Case Series

Twenty-one donors with AIS (13 women and 8 men, 18 ± 3.7 years) undergoing deformity correction surgery [42,43] were recruited. Inclusion criteria: progressive curve (>40° at diagnosis) necessitating surgical intervention. Exclusion criteria: scoliosis of different etiology. Donor characteristics are summarized in Table 1. To obtain reference gene expression levels in the same tissue types under a different condition, 6 non-age-matched surgical patients with degenerative spinal disease (4 women and 2 men, age: 63 ± 11.0 years; BMI: 24.33 ± 1.03) were recruited. Inclusion criteria: patients necessitating surgery for degenerative spinal disease and no history of scoliosis. No exclusion criteria. The study was carried out in compliance with the Helsinki declaration and approved by the local ethics committee (Prot. N. CE-AVEC 487/2022/Sper/IOR, 15 June 2022) with written informed consent.

### 4.2. Tissue Harvesting

**Figure 7:**
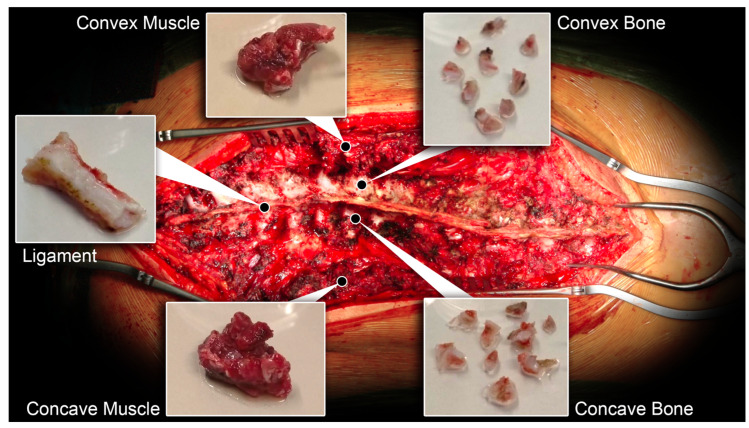
Surgical field of AIS correction showing position of bone facets (concave and convex), paravertebral muscle (concave and convex), and spinal ligament tissue sampling. A representative tissue sample of each type is also shown.

Fragments of spinal facets, paravertebral muscles, and spinal ligament from the surgical waste material of each donor were collected at the time of surgery together with a peripheral blood sample, as described [28]. To further distinguish tissues according to the side of the scoliotic curve, AIS bone and muscle samples were collected from both the convex and the concave areas. Details of the surgical technique are described in the Supplementary section, while tissue sampling in the surgical field is shown in Figure 7. Tissue samples were cut into small pieces, weighed, and then stored in RNAlater (Invitrogen, Waltham, MA, USA) at −20 °C or formalin-fixed for RNA extraction and tissue histology, respectively. White blood cells were pelleted after red blood cell lysis in 150 mM NH_4_Cl, 10 mM KHCO_3_, and 0.1 mM EDTA, pH 7.2–7.4, then stored in RNALater at −20 °C.

### 4.3. RNA Isolation

To isolate small RNA-containing total RNA (ranging in size from kilobases to 10-mers), the miRNA Isolation kit (Life Technologies, Carlsbad, CA, USA) was employed. Samples were removed from RNAlater, frozen, and pulverized, and then RNAs were extracted following the manufacturer’s instructions. Total RNA was eluted in RNase-free water, quantified, and stored at −80 °C; quality was confirmed by 260/280 and 260/230 absorbance ratios within accepted ranges for downstream analyses.

### 4.4. Gene Expression Analysis

One μg total RNA/sample was reverse-transcribed by random priming with SuperScript Vilo MasterMix (Invitrogen, Waltham, MA, USA), and then cDNAs were tested for 28 target genes and 4 housekeeping genes using a custom-designed Taqman real-time PCR array (Life Technologies, Carlsbad, CA, USA) with FAM-labeled primers and probes. The 28 genes included in the array were previously identified as promising candidate epigenetic markers of AIS progression based on comprehensive experimental evidence reported in previous works [6,24]. The 4 housekeeping genes (18S, eukaryotic 18S rRNA; B2M, beta-2-microglobulin; GAPDH, glyceraldehyde-3-phosphate dehydrogenase; and PPIA, peptidylprolyl isomerase A) were selected based on preliminary testing [28], indicating that they showed higher consistency in the tissues of interest. The final array layout is shown in Supplementary Table S2. About 10 ng cDNA/well were amplified in a final 20 μL reaction volume with Taqman Universal Master Mix II (ThermoFisher Scientific, Waltham, MA, USA) in a QuantStudio1 Real Time PCR System (ThermoFisher Scientific, Waltham, MA, USA). Reaction conditions: 10 min at 95 °C followed by 40 cycles (15 s at 95 °C, 60 s at 60 °C). Results were analyzed by the QuantStudio Design and Analysis Software v. 1.5.2 (ThermoFisher Scientific, Waltham, MA, USA), based on the relative quantification method (ΔΔC_t_) and by the RT2 Profiler PCR Array data analysis tool (Qiagen, Hilden, Germany).

miRNA-145, miRNA-151-3p, miRNA-675, and miRNA-4300 were quantified compared to the reference miRNA U6 by semi-quantitative amplification with pre-designed Taqman miRNA assays (ThermoScientific, Waltham, MA, USA). A multiplexed RT step was applied to 500 ng RNA/sample with Multiscribe Reverse Transcriptase (ThermoScientific, Waltham, MA, USA): 30 min at 16 °C, 30 min at 42 °C, 5 min at 85 °C. Template cDNAs (0.08 μL) were then amplified for each target with Taqman Universal Master Mix II (ThermoFisher Scientific, Waltham, MA, USA) in a QuantStudio1 instrument for 10 min at 95 °C, followed by 40 cycles (15 s at 95 °C, 60 s at 60 °C). Relative quantification was carried out using the ΔΔC_t_ method.

### 4.5. Histology

**Figure 8:**
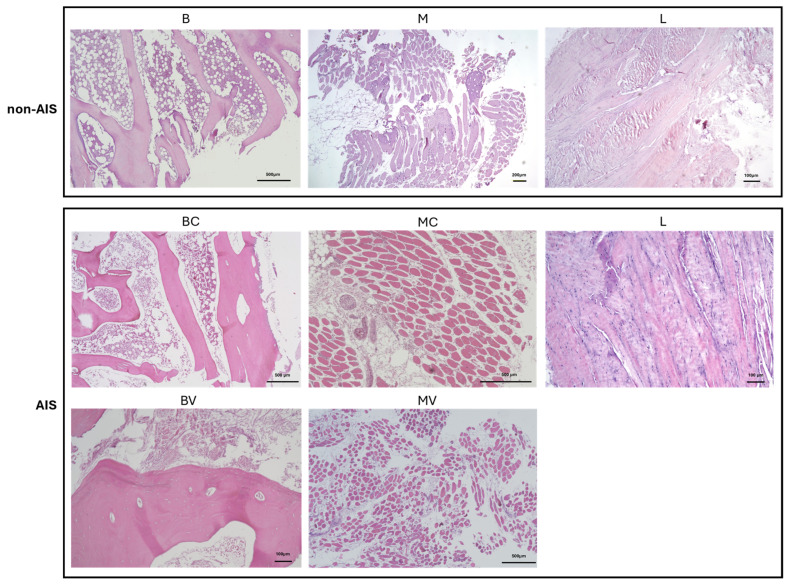
Hematoxylin–eosin staining of bone, muscle, and ligament samples from one representative donor without AIS (top) and one representative donor with AIS (bottom). B = bone (spinal facet); M = paravertebral muscle; L = spinal ligament; C = concave; V = convex.

To record representative images, tissue samples were formalin-fixed and paraffin-embedded using standard procedures, and then sections of 5 μm were stained with hematoxylin–eosin (Bioptica, Milan, Italy) and photographed (Figure 8).

### 4.6. Statistical Analysis

Data are expressed as medians, 10% to 90% percentiles, minimum and maximum values, and means ± standard deviation (SD), as appropriate.

Unsupervised hierarchical clustering of gene expression data was performed with Expression Suite Software version 1.3 (Applied Biosystems Waltham, MA, USA) using Pearson’s correlation as the distance measure, average linkage as the clustering method, and global ΔCt as the zero point for the color scale. The colors indicate deviation from the mean ΔCt value, with red representing increased gene expression and green representing decreased gene expression.

Differences in gene expression levels between AIS tissue types (bone, muscle, ligament, and peripheral blood) were analyzed using the Kruskal–Wallis test followed by Dunn’s correction for multiple comparisons.

Differences between AIS and non-AIS tissues and between the convex and concave sides of the scoliotic curve were analyzed using the Wilcoxon matched-pairs test.

The level of statistical significance was set at *p* ≤ 0.05.

Data were analyzed and graphed using GraphPad Prism software version 8.0 (GraphPad Software, La Jolla, CA, USA).

## 5. Conclusions

This study provides valuable insights into the tissue-specific gene expression patterns associated with AIS and highlights the challenges of identifying reliable blood-based biomarkers. This is, to our knowledge, the first study comparing the different musculoskeletal tissues and peripheral blood cells from the same individuals with AIS. The differential expression of specific genes and miRNAs among different AIS tissues, and between them and non-AIS samples, underscores the complexity of AIS regulation. The presented results provide the basis for further research to elucidate whether epigenetic regulatory mechanisms are actually responsible for the observed gene expression modulation, determine what these mechanisms are, and validate potential biomarkers within a restricted list of candidate genes to be explored in larger, independent cohorts.

## Figures and Tables

**Table 1:** Characteristics of donors with AIS.

Donor ID	Age at Surgery	Gender	BMI	Main Curve Localization	Menarche Status	COBB Angle	RISSER Stage
AIS 1	15	M	19	thoracic	NA	60	4
AIS 2	19	F	24	double	yes	60	5
AIS 3	20	F	23	thoracic	yes	52	5
AIS 4	19	F	21	thoracic	yes	63	5
AIS 5	23	M	22	thoracolumbar	NA	52	5
AIS 6	17	F	20	lumbar	yes	56	4
AIS 7	19	F	23	thoracic	yes	59	5
AIS 8	17	M	22	lumbar	NA	54	5
AIS 9	14	F	23	lumbar	yes	67	4
AIS 10	17	M	21	thoracic	NA	69	5
AIS 11	17	M	20	thoracic	NA	61	5
AIS 12	11	F	20	thoracic	no	45	2
AIS 13	25	F	19	thoracic	yes	81	5
AIS 14	17	F	20	thoracic	yes	57	5
AIS 15	20	M	17	thoracic	NA	86	5
AIS 16	18	F	22	thoracolumbar	yes	45	5
AIS 17	25	F	28	thoracic	yes	70	5
AIS 18	16	M	17	thoracolumbar	NA	64	4
AIS 19	12	F	25	double	yes	72	2
AIS 20	15	M	20	thoracic	NA	62	5
AIS 21	22	F	19	thoracic	yes	80	5

## Data Availability

The original contributions presented in this study are included in the article/Supplementary Material; further inquiries can be directed to the corresponding author.

## Supplementary Materials

The following supporting information can be downloaded at: https://www.mdpi.com/article/10.3390/ijms26178453/s1.

## Abbreviations

The following abbreviations are used in this manuscript:

18S	18S ribosomal RNA
PITX1	Paired-like homeodomain 1
PCDH10	Protocadherin 10
GREM1	Gremlin1
CTNNB1	Catenin beta 1
GSK3B	Glycogen synthase kinase 3 beta
LRP6	LDL receptor-related protein 6
DKK1	Dickkopf-related protein 1
GAPDH	Glyceraldehyde-3-phosphate dehydrogenase
COMP	Cartilage oligomeric matrix protein
H19	H19 imprinted maternally expressed transcript
NPY	Neuropeptide Y
FGF4	Fibroblast growth factor 4
WNT10A	Wnt family member 10A
FBN1	Fibrillin-1
MYH3	Myosin heavy chain 3
B2M	Beta-2-microglobulin
CRTC1	CREB-regulated transcription coactivator 1
ADIPOQ	Adiponectin
SOX9	SRY-box transcription factor 9
FRZB	Frizzled-related protein
AXIN1	Axin 1
FBN2	Fibrillin 2
ESR2	Estrogen receptor 2
PPIA	Peptidylprolyl isomerase A
HAS2	Hyaluronan synthase 2
ESR1	Estrogen receptor 1
APC	Adenomatous polyposis coli
FZD1	Frizzled class receptor 1
LRP5	LDL receptor-related protein 5
WNT1	Wnt family member 1
MSTN	Myostatin
