# Supplementary material for: Local Expression of Epigenetic Candidate Biomarkers of Adolescent Idiopathic Scoliosis Progression
Source: Int J Mol Sci. 2025 Aug 30;26(17):8453. doi: 10.3390/ijms26178453 (PMC12429667; doi:10.3390/ijms26178453)
Supplement: Supplementary file 1 [file ijms-26-08453-s001.zip › ijms-3801218-supplementary.pdf]

Local Expression of Epigenetic Candidate Biomarkers of Adolescent Idiopathic Scoliosis Progression

Simona Neri [1,*], Alberto Ruffilli [2,3], Elisa Assirelli [1,*], Marco Manzetti [2,3], Giovanni Viroli [2,3], Matteo Traversari [2], Marco Ialuna [2], Susanna Naldi [1], Jacopo Ciaffi [1,3], Francesco Ursini [1,3] and Cesare Faldini [2,3]

1 Medicine and Rheumatology Unit, IRCCS Istituto Ortopedico Rizzoli, 40136 Bologna, Italy; susanna.naldi@ior.it (S.N.); jacopo.ciaffi@ior.it (J.C.); francesco.ursini@ior.it (F.U.)

2 1st Orthopaedic and Traumatologic Clinic, IRCCS Istituto Ortopedico Rizzoli, 40136 Bologna, Italy; alberto.ruffilli@ior.it (A.R.); marco.manzetti@ior.it (M.M.); matteo.traversari@ior.it (M.T.); marco.ialuna@ior.it (M.I.); cesare.faldini@ior.it (C.F.)

3 Department of Biomedical and Neuromotor Sciences (DIBINEM), Alma Mater Studiorum University of Bologna, 40126 Bologna, Italy

* Correspondence: simona.neri@ior.it (S.N.); elisa.assirelli@ior.it (E.A.)

Supplementary Table S1. Expression levels (Ct values) of the four housekeeping genes (GAPDH, PPIA, 18S rRNA, and B2M) measured across all tissue samples included in the study: bone, muscle, ligament, and peripheral blood. Values are presented as mean Ct ± standard deviation. These data were used to assess the expression stability of the reference genes and to support the selection of GAPDH and PPIA for normalization in the gene expression analysis.

Tissue	GAPDH (Ct ± SD)	PPIA (Ct ± SD)	18S rRNA (Ct ± SD)	B2M (Ct ± SD)
**Bone**	24.55 ± 1.36	25.97 ± 1.50	20.61 ± 3.12	28.51 ± 4.07
**Muscle**	20.64 ± 2.18	24.99 ± 1.09	11.93 ± 2.32	23.21 ± 1.33
**Ligament**	23.95 ± 1.52	25.24 ± 1.49	15.05 ± 2.95	24.51 ± 3.10
**Peripheral blood**	22.01 ± 0.85	23.33 ± 0.88	12.67 ± 2.04	18.95 ± 1.98

Supplementary Figure S1. Local gene expression analysis in non-AIS tissues. Relative gene expression of 28 putative epigenetic markers of AIS progression in bone facets (B), intervertebral muscle (M), spinal ligament (L), and peripheral blood (PB) of 6 donors with degenerative spinal disease. Gene expression levels (relative to *PPIA* and *GAPDH* housekeepings) are shown as medians. Boxes indicate 10% to 90% percentiles, whiskers Min to Max values; dots outliers. Comparisons among different tissue types were made by Kruskal-Wallis test followed by Dunn's correction for multiple comparisons. * = p≤0.05; ** = p≤0.01 *** = p≤0.001.

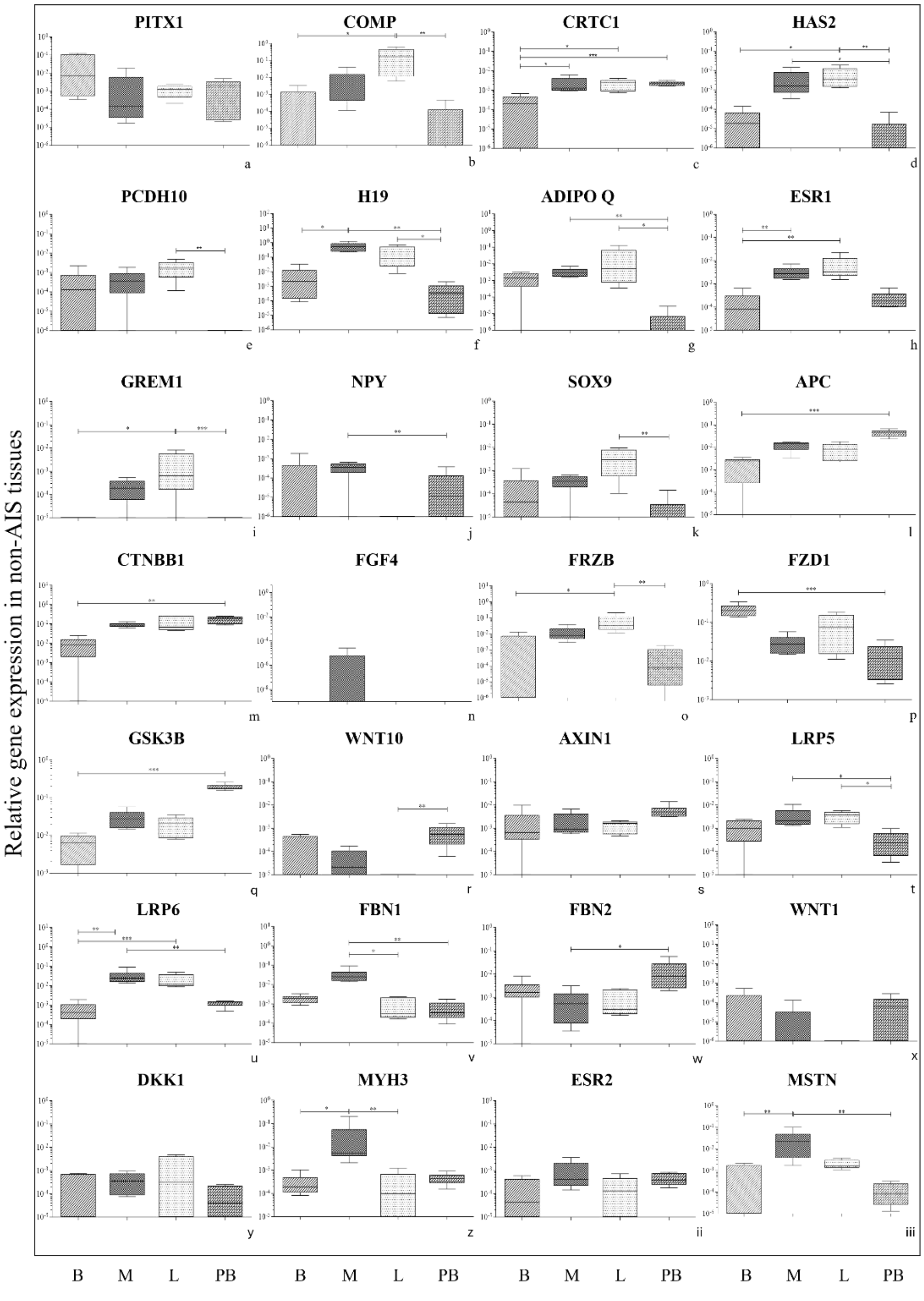

Supplementary Figure S2. Local gene expression analysis in non-AIS tissues. Relative gene expression of 4 miRNAs possibly associated to AIS progression in bone facets (B), intervertebral muscle (M), spinal ligament (L), and peripheral blood (PB) of 6 donors with degenerative spinal disease. Gene expression levels (U6 miRNA used as reference) are shown as medians. Boxes indicate 10% to 90% percentiles, whiskers Min to Max values; dots outliers. Comparisons among different tissue types were made by Kruskal-Wallis test followed by Dunn's correction for multiple comparisons. * = p≤0.05; ** = p≤0.01 *** = p≤0.001.

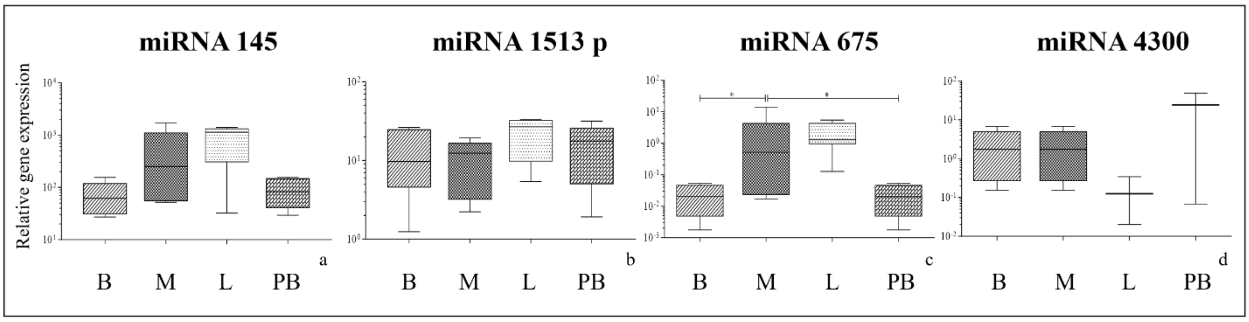

Surgical technique.

The surgical protocol addressed the unique spinal curves of each AIS patient. The patients were positioned prone on an Allen frame with continuous neuromonitoring. A subperiosteal dissection exposed the posterior spinal anatomy, followed by the placement of pedicle screws using a freehand technique and power tools, with positions verified via fluoroscopic imaging.

Cortical bone was removed to expose cancellous bone and, in the thoracic spine, the inferior articular facet was excised to guide the pedicle trajectory. Pedicle tracts were prepared with a power drill, followed by tapping. Uniplanar or polyaxial screws were used depending on the curve type.

Osteotomies were performed based on curvature type: Ponte osteotomies for thoracic curves (Ponte et al., 2018) and Smith-Petersen osteotomies (Smith-Petersen et al., 1969) for lumbar curves; more invasive osteotomies are not usually required for AIS. Two cobalt-chrome or titanium rods were shaped and placed, ensuring sagittal and coronal alignment. Corrective maneuvers can vary: posterolateral translation and apex derotation for thoracic curves; rod derotation, CD maneuver, and apical derotation for lumbar curves; a combination for double curves.

Extensors were placed at periapical levels for vertebral rotation and translation, distributing corrective forces. If needed, the rods were adjusted with in situ benders. Final steps included selective compression and distraction to close osteotomy gaps and ensure realignment. The rods were securely engaged into the tulips, providing robust and personalized spinal correction.

Control patients underwent lumbar spinal canal decompression and fusion surgery following the same steps as for scoliotic patients up to pedicle screw insertion. Subsequently, a wide decompression of the lumbar spinal canal was performed, followed by the placement of an intervertebral cage to promote fusion.

Supplementary Table S2. AIS progression epigenetic array layout. Gene symbols of the 4 housekeeping (a) and 28 target genes (b-h) are indicated. Each well of the custom array contains the lyophilized primers and probes specific for the corresponding gene. Genes are arranged in triplicate in a 96-well plate template, thus allowing for the analysis of 3 samples/plate.

a	*18S*	*GAPDH*	*B2M*	*PPIA*	*18S*	*GAPDH*	*B2M*	*PPIA*	*18S*	*GAPDH*	*B2M*	*PPIA*
b	*PITX1*	*COMP*	*CRTC1*	*HAS2*	*PITX1*	*COMP*	*CRTC1*	*HAS2*	*PITX1*	*COMP*	*CRTC1*	*HAS2*
c	*PCDH10*	*H19*	*ADIPOQ*	*ESR1*	*PCDH10*	*H19*	*ADIPOQ*	*ESR1*	*PCDH10*	*H19*	*ADIPOQ*	*ESR1*
d	*GREM1*	*NPY*	*SOX9*	*APC*	*GREM1*	*NPY*	*SOX9*	*APC*	*GREM1*	*NPY*	*SOX9*	*APC*
e	*CTNNB1*	*FGF4*	*FRZB*	*FZD1*	*CTNNB1*	*FGF4*	*FRZB*	*FZD1*	*CTNNB1*	*FGF4*	*FRZB*	*FZD1*
f	*GSK3B*	*WNT10A*	*AXIN1*	*LRP5*	*GSK3B*	*WNT10A*	*AXIN1*	*LRP5*	*GSK3B*	*WNT10A*	*AXIN1*	*LRP5*
g	*LRP6*	*FBN1*	*FBN2*	*WNT1*	*LRP6*	*FBN1*	*FBN2*	*WNT1*	*LRP6*	*FBN1*	*FBN2*	*WNT1*
h	*DKK1*	*MYH3*	*ESR2*	*MSTN*	*DKK1*	*MYH3*	*ESR2*	*MSTN*	*DKK1*	*MYH3*	*ESR2*	*MSTN*
